# Cancer Incidence and Mortality in a Tertiary Hospital in Indonesia: An 18-Year Data Review

**DOI:** 10.4314/ejhs.v33i3.15

**Published:** 2023-05

**Authors:** Prihantono, Reski Rusli, Robert Christeven, Muhammad Faruk

**Affiliations:** 1 Department of Surgery, Faculty of Medicine, Universitas Hasanuddin, Makassar, Indonesia; 2 Faculty of Medicine, Universitas Hasanuddin, Makassar, Indonesia

**Keywords:** incidence, mortality, cancer

## Abstract

**Background:**

Every population, regardless of wealth or social development, faces the major health issue of cancer. Cancer incidence and mortality differ by region and period. Thus, this study aimed to determine the characteristics, incidence, and mortality of various cancers at Dr. Wahidin Sudirohusodo Hospital, a referral center hospital in Makassar, Indonesia.

**Methods:**

This study employed a descriptive research design using secondary data recorded at Dr. Wahidin Sudirohusodo Hospital in Makassar, Indonesia, between January 2002 and December 2019.

**Results:**

We classified the 7824 cancer patients in our study into solid and non-solid cancer groups. The incidence of solid cancer (79.3%) was higher than that of non-solid cancer (20.7%), causing 1063 deaths, or 61.7%, of all cancer-related deaths. There were 6083 (77.7%) cases of cancer survival. The cancers with the highest incidence were breast cancer (1008 cases [12.9%]), leukemia (683 cases [8.7%]), and cervical cancer (631 cases [8.1%]). Breast, cervical, and ovarian cancers were the most frequent cancers in female patients. Leukemia was the most frequent cancer in male patients, followed by colorectal and liver cancers.

**Conclusions:**

A region-based statistical record of cancer incidence and mortality is vital and useful to prioritizing cancer treatment at a given time.

## Introduction

Cancer has had an unequal and unfair impact across the world. In 2018, globally, 9.6 million individuals died from cancer, which affected approximately 18.1 million individuals. More than two-thirds of global cancer cases occur in low- and middle-income countries, a rate that will nearly triple by 2040 (1). In males, the prostate, lung and bronchus, colon and rectum, and urinary bladder are the organs most frequently affected by cancer, while in females, the breast, lung and bronchi, colon and rectum, uterine corpus, and thyroid are the organs most frequently affected by cancer (2). Significant portions of all cancers in males and females affect the prostate and breast, respectively. In children, blood, brain, and lymph node cancers are the most frequent cancers (in decreasing order) (3).

Cancer mortality profiles in Indonesia indicate that the most new cancers are breast (43.3%), prostate (30.7%), and lung (23.1%) cancers. Baseline Health Research (BHR) data showed an increase in cancer prevalence in Indonesia from 1.4% in 2013 to 1.49% in 2018. Gorontalo Province had the greatest increase, from 0.2% in 2013 to 2.44% in 2018, although significant increases also occurred in Central Sulawesi Province, from 0.9% in 2013 to 2.2% in 2018. Six provinces—Jambi, Bengkulu, East Kalimantan, South Sulawesi, Maluku, and North Maluku— showed decreases in prevalence. Cancer prevalence is relatively high in DI Yogyakarta Province compared to the other provinces, going from 4.1% in 2013 to 4.86% in 2018 (4). This study aimed to determine the characteristics, incidence, and mortality of various cancers at Dr. Wahidin Sudirohusodo Hospital, a referral center hospital in Eastern Indonesia.

## Patients and Methods

This study used a descriptive research design. The characteristics and incidences of cancers were ascertained based on secondary data recorded in medical records at Dr. Wahidin Sudirohusodo Hospital, a referral center hospital in Makassar, Indonesia, between January 2002 and December 2019. The participants of the study included all new cancer patients treated at this hospital in the same time period. This study obtained ethical approval from the Ethical Committee of the Faculty of Medicine, Universitas Hasanuddin, Makassar, South Sulawesi, Indonesia (No. 823/UN4.6.4.5.31/PP.36/2022).

A total sampling approach was used, in which all patients who met the research criteria were included; the exclusion criterion was new cases with secondary tumors. Solid tumors included cancers of the breast, cervix, colon, ovary, or other tissues, while nonsolid tumors are commonly known as blood cancer (e.g., leukemia or lymphoma).

The data were processed and analyzed using Microsoft Excel (Seattle, WA, USA) and the Statistical Package for Social Sciences (SPSS) version 25.0 (IBM Corp., Armonk, NY, USA). The data are presented as distribution graphs, with explanations arranged in narrative form, and are grouped according to the research objectives.

**Ethical Approval:** The ethical approval of this study was granted from Ethical Committee, Faculty of Medicine, Universitas Hasanuddin, Makassar, Indonesia (number: 823/UN4.6.4.5.31/PP.36/2022).

## Results

The investigation of medical record data identified 8092 new cancer cases that met the inclusion criteria during the study period. However, 268 cases were diagnosed as secondary malignant tumors, so the total number of cases meeting both the inclusion and exclusion criteria was 7824.

**Figure 1:**
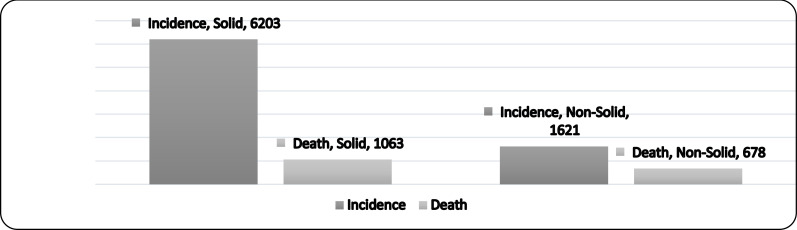
Incidence and mortality by cancer type.

**Incidence and mortality by cancer type**: The incidence of solid cancers was 6203 cases (79.3%), and these resulted in 1063 deaths (61.7% of all cancer-related deaths). In contrast, the incidence of non-solid cancers was 1621 cases (20.7%), resulting in 678 deaths (38.3% of all cancer-related deaths; Figure 1).

**Figure 2:**
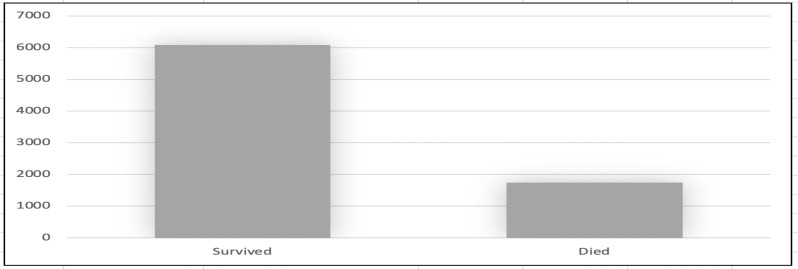
Mortality by cancer type.

**The distribution of cancer incidence by mortality**: Data from the 7824 patients' medical records showed that 6083 (77.7%) survived, while 1741 (22.3%) died (Figure 2).

**Cancer incidence by sex**: Cancer was more frequently found in females (4485 cases [57.3%]) than in males (3339 cases [42.7%]).

**Figure 3:**
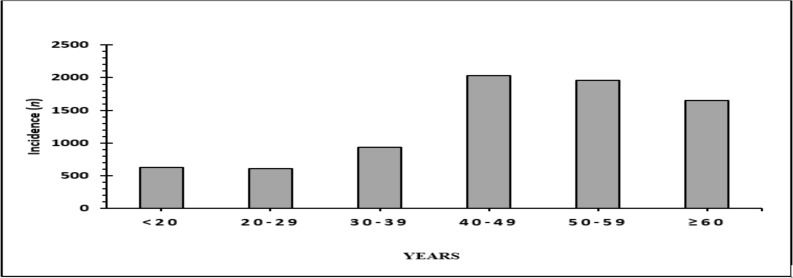
Cancer incidence by age

**Cancer incidence by age**: The youngest cancer patient in this study was six months and the oldest was 93 years, with a mean age of 45.4 ± 0.2 years. Most patients aged 40–49 years (2035 cases [26%]), followed by 50–59 years (1962 cases [25.1%]), 60 years or older (1653 cases [21.1%]), 30–39 years (935 cases [12%]), younger than 20 years (633 cases [8.1%]), and 20–29 years (606 cases [7.7%]). Therefore, the most frequent age for cancer diagnosis was the fifth decade (Figure 3).

**Figure 4:**
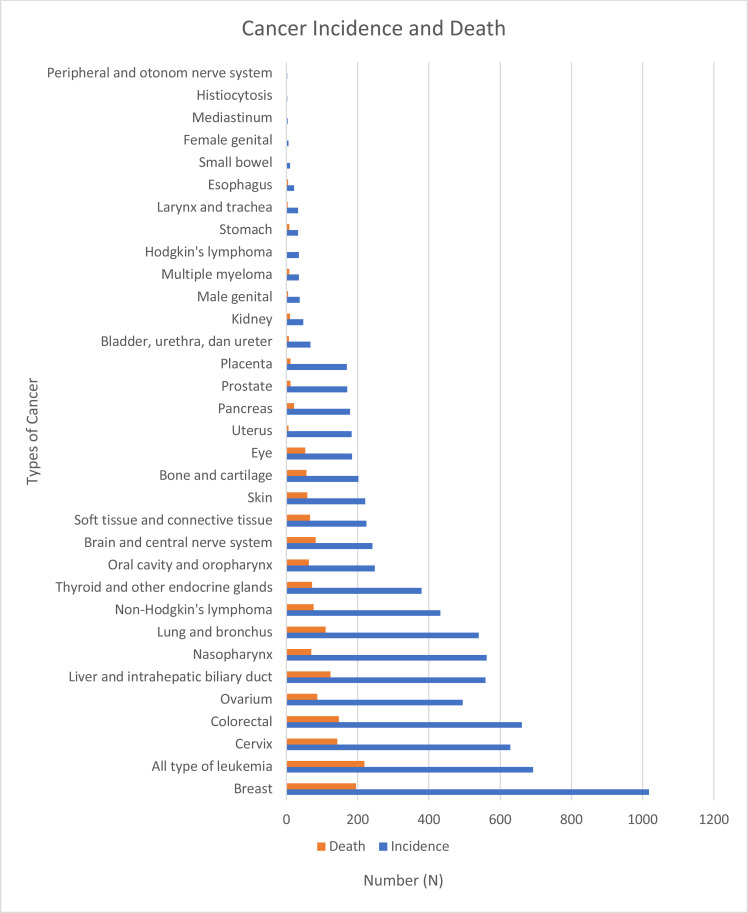
Incidence and mortality by cancer location

**Incidence and mortality by cancer location**: The cancers with the highest incidences were breast cancer (1008 cases [12.9%]), leukemia (683 cases [8.7%]), and cervical cancer (631 cases [8.1%]), followed by colorectal (551 cases [7.0%]) and ovarian (496 cases [6.3%]) cancers. The cancers with the lowest incidences were histiocytosis and cancer of the peripheral and autonomic nervous systems, which had three cases (0.01%) each (Figure 4). Leukemia caused the most deaths (219 cases [12.7%]), followed by breast (198 cases [11.4%]) and colorectal (147 cases [8.5%]) cancers. Cancer of the peripheral and autonomic nervous systems caused no deaths.

**Figure 5:**
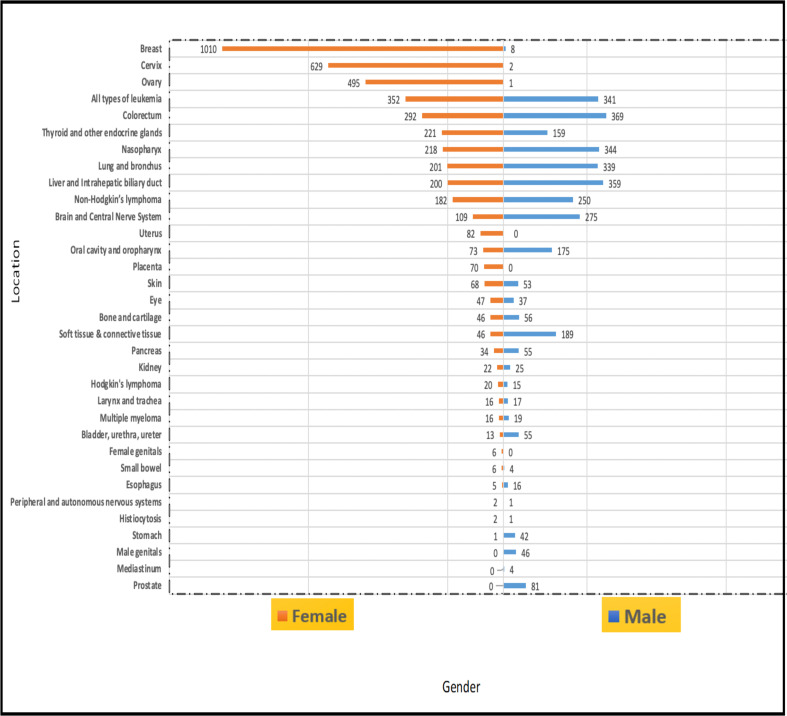
Incidences of cancer by location and sex.

**Cancer location by sex**: In females, breast (1010 cases [15.6%]), cervical (629 cases [9.1%]), and ovarian (495 cases [6.8%]) cancers were the most common cancers, followed by leukemia (352 cases [4.3%]) and nasopharyngeal cancer (218 cases [2.0%]). In males, colorectal cancer (369 cases [5.7%]) was the most common, followed by liver and intrahepatic biliary duct cancer (359 cases [4.7%]), nasopharyngeal cancer (344 cases [4.4%]), and leukemia cancers (341 cases [4.1%]). The complete data are shown in Figure 5.

**Figure 6:**
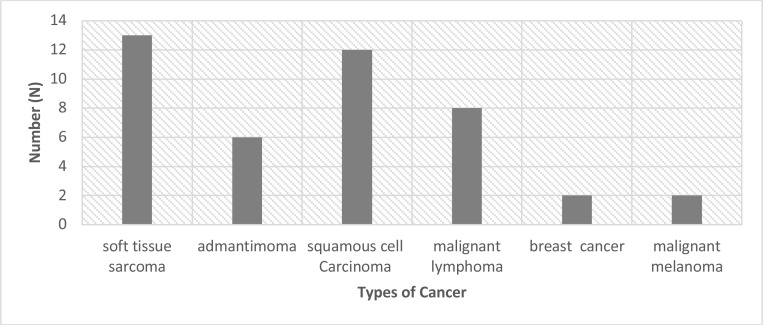
Cancer incidences in pediatric patients.

**Cancer types in pediatric patients**: Among the 7824 cancer cases, 43 (0.55%) were in children aged 18 years or younger. Soft tissue sarcoma was the most common cancer in the pediatric population (13 cases [30.2%]), and malignant lymphoma was the only non-solid tumor identified in this population (2 cases [18.6%]) (Figure 6).

## Discussion

The study focused on the cancer characteristics of incidence (number of new cases), mortality, sex, age, type, and location of cancer incidence in eastern Indonesia.

Our data included 6083 (77.7%) survival cases and 1741 (22.3%) death cases. Globally, in 2020, there were approximately 10.2 million cancer-related deaths (9.9 million excluding non-melanoma skin cancer) and 19.3 million new cancer cases (excluding non-melanoma skin cancer) (5), making the death rate 51.81%. According to the 2020 Global Cancer Observatory (GLOBOCAN) data, the number of new cancer cases in Indonesia was 396,914, and the number of cancer-related deaths was 234,511 (59.08%) (6).

While it varies considerably by region, in 2020 the global incidence of all malignancies combined was 19% higher in males (222 per 100,000) than in females (186 per 100,000). Huang et al. examined cancer populations in Asia and found that cancer incidence had increased in females (7). Among males, incidence rates varied five-fold, from 494.2 per 100,000 in Australia/New Zealand to 100.6 per 100,000 in western Africa; among females, incidence rates varied nearly four-fold, from 405.2 per 100,000 in Australia/New Zealand to 102.5 per 100,000 in south-central Asia. These variations can reflect differences in exposure to risk factors and associated cancers (cancer mix) as well as barriers to high-quality cancer prevention and early detection. For example, the higher overall incidence in Australia/New Zealand is partly caused by an elevated risk for non-melanoma skin cancer, since most of the population is both light-skinned and prone to excessive sun exposure (5). According to the 2020 GLOBOCAN data, the number of new cancer cases in Indonesia was 183,368 in males and 213,546 in females (6). In this study, cancer incidence was higher in females (4485 cases [57.3%]) than in males (3339 cases [42.7%]).

According to data from Indonesia's Hospital-Based Cancer Registry collected between 2008 and 2012, most patients aged > 39 years (12,438 [68.3%]), followed by 20–39 years (3,971 [21.8%]) and 0–19 years (1,807 [9.9%]) (8). These data are consistent with our study, which also showed that most new cancer cases were diagnosed in patients aged 40 years or older (5,650 [72.2%]): most new cancer patients were aged 40–49 years (2035 [26.4%]), followed by 50–59 years (1962 [25.1%]). Moreover, the age range with the fewest new cancer patients was 20–29 years (606 [5.3%]).

The United Kingdom Cancer Research data indicates that age-specific incidences increase dramatically at approximately 55–59 years, with males and females aged 85–89 years having the highest incidence. More than half (54%) of all new cancer cases occur in adults aged 50–74 years, and 36% occur in adults aged ≥75 years (In both age categories, females are slightly less likely to develop cancer than males). Since there are more individuals aged 50–74 years than individuals aged ≥75 years in the population, the number of cancer cases is higher in those aged 50–74 years, but incidence rates are higher in those aged ≥75 years (9).

We classified cases into solid and non-solid cancer groups. In our study, the solid cancer incidence was 6203 cases (79.3%) resulting in 1063 deaths (61.7% of all cancer-related deaths), while the non-solid cancer incidence was 1621 cases (20.7%) resulting in 678 deaths (38.3%). No previous study reported incidence rates for all solid and non-solid cancers in Indonesia. In our study, breast cancer, a solid tumor, was the most common cancer (1008 cases [12.9%]); however, leukemia was the most frequent cause of cancer-related death (219 cases [12.7%]). According to the 2020 the GLOBOCAN data, the most frequent cancer in Indonesia and worldwide is breast cancer, while the greatest mortality rates are for lung, colorectal, liver, and stomach cancers (5,6). Breast cancer is also the most common solid cancer in Africa (10).

Breast (1010 cases [15.6%]), cervical (629 cases [9.1%]), and ovarian (495 cases [6.8%]) cancers were the most common cancers affecting females in our study. In contrast, colorectal cancer was the most common in males (369 cases [5.7%]), followed by liver and intrahepatic biliary duct cancer (359 cases [4.7%]). According to the 2020 GLOBOCAN data, lung cancer was the most common cancer and the leading cause of cancer-related death in males in 2021, followed by prostate, colorectal, and liver cancers in incidence rates and colorectal and liver cancers in mortality rates. In females, colorectal and lung cancers have the highest incidences, and lung and colorectal cancers have the highest mortalities (5); thereafter, breast cancer is the most frequently diagnosed cancer and the most common cause of cancer-related death (5,11).

More than 80% of pediatric cancer cases diagnosed are in low- and middle-income countries (LMICs), where access to detection and treatment is frequently poor. According to Ward et al. (12), there were 397,000 childhood cancer cases worldwide in 2015, but only 224,000 were diagnosed; they reported that 172,000 of these 397,000 cases went undiagnosed worldwide—with significant regional variance, ranging from 3% in western Europe and north America (120 of 4300 cases each) to 57% in western Africa (43,000 of 76,000 cases). In that study, the overall fraction of undiagnosed cases in southern Asia (including southeastern and south-central Asia) was estimated to be 49% (67,000 of 137,000) (12). In our study, we identified 43 (0.55%) cases of cancer in pediatric patients (aged <18 years), which is a relatively small number. In Indonesia, early detection of childhood cancer is still relatively rare; knowledge about the signs and symptoms of childhood cancer remains relatively poor in developing countries, including Indonesia. Our results are similar with those of Endalamaw et al., who found the prevalence of pediatric cancer among 1257 subjects to be 0.8% (13).

In our study, sarcoma and squamous cell carcinoma were the most common pediatric cancers. This result is relatively unique, since soft tissue sarcomas are rare in children: the incidence of soft tissue sarcomas in children aged 20 years or younger is 11.0 per million, representing 7.4% of cancer cases in this age group (14). Leukemia is the most common cancer in childhood worldwide (15). However, Sharma et al. found that the most frequent pediatric cancer in India was central nervous tumors (25.7%) (16), indicating that demographic differences affect the childhood prevalence rates of different cancers.

In conclusion, cancer incidence and mortality can vary dynamically according to time and region; thus, worldwide and country-specific cancer statistics do not always represent cancer incidence and mortality for a given region. An area-based statistical record of cancer incidence and mortality is useful to prioritizing cancer treatment at a given time.
